# Perceived and observed biases within scientific communities: a case study in movement ecology

**DOI:** 10.1098/rspb.2025.0679

**Published:** 2025-07-23

**Authors:** Allison K. Shaw, Leila Fouda, Stefano Mezzini, Dongmin Kim, Nilanjan Chatterjee, David Wolfson, Briana Abrahms, Nina Attias, Christine E. Beardsworth, Roxanne S. Beltran, Sandra A. Binning, Kayla M. Blincow, Ying-Chi Chan, Emanuel A. Fronhofer, Arne Hegemann, Edward R. Hurme, Fabiola Iannarilli, Julie B. Kellner, Karen D. McCoy, Kasim Rafiq, Marjo Saastamoinen, Ana M. M. Sequeira, Mitchell W. Serota, Petra Sumasgutner, Yun Tao, Martha Torstenson, Scott W. Yanco, Kristina B. Beck, Michael G. Bertram, Larissa T. Beumer, Maja Bradarić, Jeanne Clermont, Diego Ellis-Soto, Monika Faltusová, John Fieberg, Richard J. Hall, Andrea Kölzsch, Sandra Lai, Larisa Lee-Cruz, Matthias-Claudio Loretto, Alexandra Loveridge, Marcus Michelangeli, Thomas Müller, Louise Riotte-Lambert, Nir Sapir, Martina Scacco, Claire S. Teitelbaum, Francesca Cagnacci

**Affiliations:** ^1^ Department of Ecology, Evolution, and Behavior, University of Minnesota Twin Cities, Saint Paul, MN, USA; ^2^ Department of Biological Sciences, University of New Brunswick, Saint John, New Brunswick, Canada; ^3^ Center for Biodiversity and Global Change, Yale University, New Haven, CT, USA; ^4^ Department of Ecology and Evolutionary Biology, Yale University, New Haven, CT, USA; ^5^ Okanagan Institute for Biodiversity, Resilience, and Ecosystem Services, The University of British Columbia Okanagan, Kelowna, British Columbia, Canada; ^6^ Department of Biology, The University of British Columbia Okanagan, Kelowna, British Columbia, Canada; ^7^ Department of Organismic and Evolutionary Biology, Harvard University, Cambridge, MA, USA; ^8^ Department of Fisheries, Wildlife, and Conservation Biology, University of Minnesota Twin Cities, Saint Paul, MN, USA; ^9^ Senckenberg Biodiversity and Climate Research Centre, Frankfurt, Hessen, Germany; ^10^ Department of Biology, University of Washington, Seattle, WA, USA; ^11^ Instituto de Conservação de Animais SIlvestres, Campo Grande, Brazil; ^12^ Center for Latin American Studies, University of Florida, Gainesville, FL, USA; ^13^ School of Biological and Environmental Sciences, Liverpool John Moores University, Liverpool, UK; ^14^ Ecology and Evolutionary Biology, University of California Santa Cruz, Santa Cruz, CA, USA; ^15^ Département de Sciences Biologiques, Université de Montréal, Montréal, Québec, Canada; ^16^ College of Science and Mathematics, University of the Virgin Islands, Saint Thomas, Virgin Islands; ^17^ Swiss Ornithological Institute, Sempach, Switzerland; ^18^ ISEM, University of Montpellier-CNRS-IRD, Montpellier, Occitanie, France; ^19^ Department of Biology, Lund University, Lund, Sweden; ^20^ Department of Migration, Max Planck Institute of Animal Behavior, Radolfzell, Baden-Württemberg, Germany; ^21^ Advanced Study of Collective Behaviour, University of Konstanz, Konstanz, Baden-Württemberg, Germany; ^22^ Biology Department, Woods Hole Oceanographic Institution, Woods Hole, MA, USA; ^23^ MiVEGEC, University of Montpellier-CNRS-IRD, Montpellier, Occitanie, France; ^24^ Organismal and Evolutionary Biology Research Programme, Faculty of Biological and Environmental Sciences, University of Helsinki, Helsinki, Finland; ^25^ Division of Ecology and Evolution, Australian National University, Canberra, Australian Capital Territory, Australia; ^26^ UWA Oceans Institute and the School of Biological Sciences, The University of Western Australia, Perth, Australia; ^27^ Environmental Science, Policy, and Management, University of California Berkeley, Berkeley, CA, USA; ^28^ Konrad Lorenz Research Center (KLF), University of Vienna, Vienna, Austria; ^29^ Institute of Bioinformatics, University of Georgia, Athens, GA, USA; ^30^ School for Environment and Sustainability, University of Michigan, Ann Arbor, MI, USA; ^31^ Migratory Bird Center, Smithsonian’s National Zoo and Conservation Biology Institute, Washington, DC, USA; ^32^ Department of Wildlife, Fish, and Environmental Studies, Swedish University of Agricultural Sciences, Umeå, Västerbotten, Sweden; ^33^ Department of Zoology, Stockholm University, Stockholm, Sweden; ^34^ School of Biological Sciences, Monash University, Melbourne, Victoria, Australia; ^35^ The University Centre in Svalbard, Longyearbyen, Norway; ^36^ Institute for biodiversity and ecosystem dynamics, University of Amsterdam, Amsterdam, Noord-Holland, The Netherlands; ^37^ Département de biologie, Université de Sherbrooke, Sherbrooke, Québec, Canada; ^38^ Faculty of Forestry and Wood Sciences, Czech University of Life Sciences, Suchdol, Czech Republic; ^39^ Odum School of Ecology, University of Georgia, Athens, GA, USA; ^40^ Department of Infectious Diseases, University of Georgia, Athens, GA, USA; ^41^ Department of Ecology (RIBES), Radboud University, Nijmegen, Netherlands; ^42^ Department of Biology, University of Oxford, Oxford, UK; ^43^ UMR TETIS, French Agricultural Research Centre for International Development, Montpellier, France; ^44^ Research Institute of Wildlife Ecology, Department of Interdisciplinary Life Sciences, University of Veterinary Medicine Vienna, Vienna, Austria; ^45^ The Marine Biological Association, Plymouth, UK; ^46^ National Oceanography Centre, Southampton, UK; ^47^ School of Environment and Science, Griffith University, Brisbane, Queensland, Australia; ^48^ Department of Biological Sciences, Goethe University, Frankfurt, Germany; ^49^ CEFE, CNRS, University of Montpellier-EPHE-IRD, Montpellier, Occitanie, France; ^50^ Department of Evolutionary and Environmental Biology and Institute of Evolution, University of Haifa, Haifa, Israel; ^51^ NASA Ames Research Center, Moffett Field, CA, USA; ^52^ Bay Area Environmental Research Institute, Moffett Field, CA, USA; ^53^ Animal Ecology Unit, Research and Innovation Centre, Fondazione Edmund Mach, San Michele all'Adige, Trento, Italy; ^54^ National Biodiversity Future Centre, Palermo, Italy

**Keywords:** academic conference, diversity, equity, journal authorship, parachute science, representation

## Abstract

Who conducts biological research, where they do it and how results are disseminated vary among geographies and identities. Identifying and documenting these forms of bias by research communities is a critical step towards addressing them. We documented perceived and observed biases in movement ecology, a rapidly expanding sub-discipline of biology, which is strongly underpinned by fieldwork and technology use. We surveyed attendees before an international conference to assess a baseline within-discipline perceived bias (uninformed perceived bias). We analysed geographic patterns in *Movement Ecology* articles, finding discrepancies between the country of the authors’ affiliation and study site location, related to national economics. We analysed race-gender identities of USA biology researchers (the closest to our sub-discipline with data available), finding that they differed from national demographics. Finally, we discussed the quantitatively observed bias at the conference, to assess within-discipline perceived bias informed with observational data (informed perceived bias). Although the survey indicated most conference participants as bias-aware, conversations only covered a subset of biases. We discuss potential causes of bias (parachute-science, fieldwork accessibility), solutions and the need to evaluate mitigatory action effectiveness. Undertaking data-driven analysis of bias within sub-disciplines can help identify specific barriers and move towards the inclusion of a greater diversity of participants in the scientific process.

## 1. Introduction

Biases (systematic distortions with respect to the distribution of a reference population) universally affect the production and dissemination of scientific research. These biases include the topics prioritized for funding [1,2] and publication [3,4], which papers are cited [5–7], the language in which results are communicated [8,9] and the identities of authors in the peer-reviewed literature, especially in high-impact international journals [10,11]. Indeed, the composition of the scientific community itself reflects long-standing biases in terms of gender [12–15], race and ethnicity [16,17], socioeconomic status and family education level [18,19]. Beyond ethical concerns, these biases limit scientific progress and constrain insights and innovations [20]. For example, a lack of researcher gender diversity can limit the research questions and topics addressed [21]. Cultural biases can also impact interpretation of science [22]. Indigenous knowledge can provide critical information like traditional sustainable ways of living [23], or historical animal migratory corridors that have been lost [24]. Omitting these perspectives can lead to knowledge gaps and perpetuate inequalities that impact science policy decision-making [25]. Within biology, biases also include the geographic locations of research [7,26] and taxonomic group(s) studied [27–29].

There is an urgent need for the scientific community to document how these biases develop, persist and change, in order to work proactively towards the goal of broadening equitable participation [30]. Yet, the range of these biases is rarely quantified within specific disciplines. Only studying biases at a broad disciplinary level (e.g. across all of biology) can risk overlooking important sub-discipline-specific factors that are needed to formulate targeted actions to rectify inequities. Aiming to solve issues at the sub-discipline level can be more approachable. Often in biology, there are sub-discipline-specific communities that can be succinctly targeted to improve their participation on shorter time scales than an entire disciplinary field. Case studies within sub-disciplines can also illustrate concrete examples of biases and suggest potential paths forward [31,32]. Within a sub-discipline, examining bias at different scales (e.g. clarifying if biases manifest within or among countries [9] or looking into gender equality in the discipline [32,33]) helps shape the scope and nature of potential solutions. Furthermore, determining the extent to which researchers in a sub-discipline discern biases (perceived bias) and the degree to which biases manifest in the quantifiable activities of that discipline (observed bias) represent critical initial steps in identifying potential solutions.

This paper uses movement ecology as a case study of sub-discipline-specific bias. Movement ecology [29,34,35] is an emerging sub-discipline of biology that is increasingly represented in high-impact journals [36–38] and is the subject of a recently launched (2013) journal (*Movement Ecology*), and multiple international conferences. The recent growth of this sub-discipline emphasizes the need to critically examine and address its embedded biases. The fieldwork-intensive nature of movement ecology often requires expensive technology, large datasets, remote travel, specialized training and computational skills and resources, all of which can magnify extant biases.

Here, we (a biased sample of the movement ecological community; see positionality statement in electronic supplementary material, S1) describe biases present within movement ecology. Our overall goals are to start a conversation on rectifying bias in our community and to present a case study that can be used by other sub-disciplines within biology. We use four approaches to consider both perceived biases (looking at uninformed and informed perspectives) and observed bias (considering two forms of bias with respect to our community). First, we quantify perceived biases among attendees of a conference in our sub-discipline via a survey (including all forms of bias). Next, to quantify the extent to which biases manifest, we assess two forms of bias: geographic bias and race-gender identities. For the first, we quantified the geography of authors’ institutions and of study areas (i.e. where research is conducted) for articles published in the sub-discipline’s primary journal. For the second, we could not find a way to quantify race-gender identities within our sub-discipline without adding bias in our assumptions about personal identities and how they depend on cultural context (but see alternative approaches in [32]). So instead, we quantified representation in the broader discipline of biology with respect to self-identified race and gender identities of academic biologists in one country only (United States), where these data were available. We discussed these three findings (uninformed perceived bias via survey results, and the two forms of observed bias) at a conference on movement ecology, within a specific session organized on this topic, where conversations could span all forms of bias. Here, we summarize these discussions as perceived bias *informed* by observed bias data. We then contextualized the results within our sub-discipline, identifying factors shaping the emergence of specific biases. We conclude by discussing potential solutions and assessing if any have been (successfully) implemented.

## 2. Methods

### (a) Uninformed perceived bias: pre-conference survey

To quantify perceived bias within the movement ecology community, we conducted an anonymous email survey of attendees registered for an international conference in the sub-discipline. The conference, held in Tuscany, Italy in May 2023, was the third edition of a thematic conference series on movement ecology of animals that primarily addresses an audience of specialists in the sub-discipline (the conference management board does not allow us to name it explicitly). The attendees of these conferences are a mixture of invited speakers and selected poster presenters, with diversity being a criterion of selection alongside scientific excellence as per the conference series guidelines. The conference participation is capped at 200 individuals; in 2023 it was attended by 196 people from 24 countries on 5 continents (electronic supplementary material, figure S1). The conference fee exceeded US$1300 (including accommodation and food, but not transportation). Some of this cost was offset for some participants by conference grant funding.

A.K.S. and F.C. designed and distributed the survey, using a mixed approach with short-answer questions (allowing open-ended responses) and multiple-choice questions (easier to analyse quantitatively) (electronic supplementary material, S2). Survey takers answered to what extent they perceived bias in the community (Q1), what the main sources of bias were (Q2 short answer, Q3 multiple choice), which experiences their answers were based on (Q4), how the community could become less biased (Q5), and who should be responsible for addressing bias in the community (Q6). To generate the multiple choice options for Q3, we requested input from researchers of different backgrounds who work on movement ecology but were not attending the conference. A total of 135 survey responses were collected between 26 April and 24 May 2023, prior to the conference. Closed-ended survey questions were analysed by plotting the distribution of responses for Q1, Q4 and Q6, separated out by the response to Q3. Open-ended survey questions were analysed by reading responses and creating a code (i.e. label) for each idea present in the response (codes were not determined *a priori*). After coding, we looked for emergent themes, and grouped codes by theme. Coding and theme analysis was done by A.K.S.

### (b) Observed bias

#### (i) Geographic bias for authors and research: *Movement Ecology* journal articles

To provide an example of observed bias in research and researchers’ geographies within the sub-discipline, we analysed geographic patterns of authors’ institutional affiliation and study location for articles published in the open-access, flagship journal of our sub-discipline, *Movement Ecology*. We downloaded article information via Scopus on 21 January 2023, yielding 370 articles published between the journal’s inception in 2013 to the data download date. For each article, we extracted the first country of affiliation listed (i.e. first affiliation if multiple were listed) and the number of countries of affiliation for the first and corresponding authors. We quantified the number of times each country appeared as the first country of affiliation (first author or corresponding author) for all 370 articles. For each article, we determined whether the primary data used in the article were newly collected for the study and, if yes, the country of data collection. For studies that tracked animals that crossed international borders, we only included the country/territory where the trackers were deployed. We determined whether or not any author on the article had any affiliation in the country of data collection. Of the 370 articles, 266 were considered to have collected new data, while the other 104 were excluded from this part of the analysis because they were corrections, review papers, meta-analyses, theory or used simulated or previously published data. We used country/territory names as they appear on the World Bank list [39] and World Bank data [40] for the most recently available yearly gross domestic product (GDP) data for each country. We estimated the effect of GDP on publications for both yearly GDP versus number of publications and per-capita GDP versus per-capita publications, using a generalized additive model (see electronic supplementary material, S1). Finally, we grouped countries into regions and compared each study from the institutional affiliation to the location of fieldwork, based on 2022 gross national income (GNI) from the World Bank Atlas [41] (see electronic supplementary material, S1 ).

#### (ii) Race and gender bias: USA biology researchers

To provide an example of observed bias by race/ethnicity and gender across career stages of academia as compared to the general public, we used demographic representation data for the United States of America (USA). We chose the USA because it is a country that collects and publicly disseminates this self-assessed demographic data on a national scale. Although it was not possible to have an unbiased (i.e. self-identified) representation of race/ethnicity and gender within the sub-discipline of movement ecology and across geographies, our choice considers data from the country with the most first authors of *Movement Ecology* articles (*n* = 120 of 370). We used 2021 data for graduate students, postdoctoral researchers and faculty, and census data for the USA general population for 2019. For each group, we calculated the proportion of individuals who identified as each gender-and-race combination (hereafter ‘race-gender group’) and compared this to the proportion of each race-gender group across the censused USA population, to get relative representation (see electronic supplementary material, S1 and table S1).

### (c) Perceived bias informed by observed bias data: conference discussion

Finally, to assess observation-informed perceived bias, results of both the survey and the observed bias approaches were presented and discussed at an in-person conference session on inclusion and barriers to inclusivity. The day before the session, all conference attendees were provided a handout summarizing the findings of the survey, the geography bias data and the race-ethnicity bias data (see handout in electronic supplementary material, S3) and were invited to attend the discussion. Of the 196 conference attendees, 105 participated in the discussion. Attendees were split into 10 groups of 8−12 people. The discussion leaders (F.C. and A.K.S.) described the goals of the session, went over the handout, and provided attendees with prompts to discuss in their groups. Each group identified a scribe to record notes. The groups discussed the prompts individually, and then reported back to the broader group. Notes from each group were compiled and summarized by the discussion leaders. Everyone who participated in the discussion was invited to be a potential co-author on this publication (see positionality statement in electronic supplementary material, S1).

## 3. Results

### (a) Uninformed perceived bias: pre-conference survey

#### (i) Frequency and sources of bias

**Figure 1.**
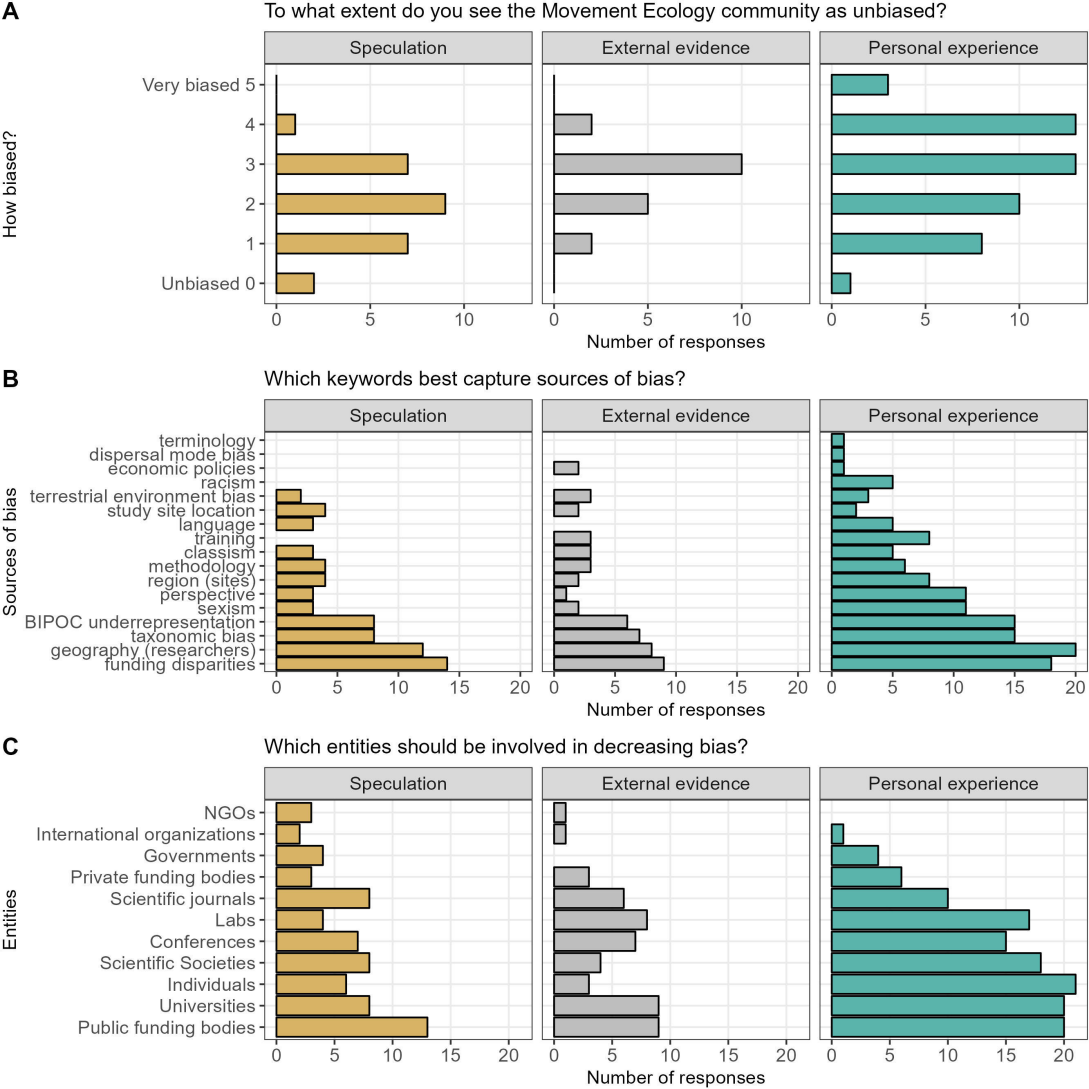
Survey responses split by each individual’s report of the main source of evidence of bias (only one could be selected): speculation (yellow), external evidence such as readings (grey) and personal experience (green). Questions covered (A) bias within the movement ecology community, (B) sources of bias and (C) entities that should be involved in decreasing bias.

We received survey responses from 135 out of approximately 200 distributed surveys (although not all respondents answered every question). Nearly all those who answered question Q1 (‘To what extent do you see the Movement Ecology community as unbiased?’; see survey in electronic supplementary material, S2), believed that there was bias present within the movement ecology community (96.8%, *n* = 90; figure 1A). The majority of respondents rated this bias as low (44.1%, *n* = 41, scores 1−2), whereas 20.4% (*n* = 19) of respondents perceived bias as high (scores of 4−5). These patterns largely correlated with the respondent’s own experience of bias as identified in question Q3 (‘What is your previous answer *mainly* based on?’). Specifically, 55.6% (*n* = 25) of individuals with no direct experience of bias perceived little to no bias within the community, compared to 39.6% (*n* = 19) of individuals with personal experience of bias. Conversely, only 6.7% (*n* = 3) of individuals with no direct experience of bias perceived the community as highly biased, compared to 33.3% (*n* = 16) of those with personal experience.

Survey respondents’ own experience of bias influenced not only their perceived frequency of bias but also the sources of bias. In response to the open-ended question Q2 (‘What do you think the main sources of bias are, if any?’), the most commonly reported answers were biases associated with identity of researchers (e.g. gender, race, nationality) (electronic supplementary material, table S2). Other answers related to what is studied (e.g. study species, region), how it is studied (e.g. technology, methodology), and how our community works (e.g. access to training and opportunities). In response to a closed-ended question Q4 (‘Which of the following keywords best capture sources of bias in the Movement Ecology community?’), individuals with personal experience of bias reported the greatest number of perceived sources of bias (*n* = 17) relative to those with no direct experience (*n* = 13) (figure 1B). Overall, ‘funding disparities’, ‘geographic concentration of researchers’, ‘taxonomic bias’ and ‘BIPOC [Black, Indigenous, and other People of Colour] underrepresentation’ were consistently reported as key sources of bias, accounting for 55.2% (*n* = 88) of the keywords best capturing the sources of bias (see electronic supplementary material, S3 for full list of options in the survey). In contrast, there were differences in perceived sources across groups with different experiences of bias. For example, explicit categories of discrimination—‘sexism’, ‘racism’ and ‘classism’—accounted for 15.6% (*n* = 21) of keywords for individuals with direct experience of bias, ranking 5th, 7th and 8th, respectively. However, ‘sexism’ and ‘classism’ were less perceived as sources of bias by respondents with no personal experience of bias, with ‘racism’ not perceived as one of the top three issues. Together, these data highlight the prominent role of personal experience in dictating the perception of frequency and source of bias within a research community.

#### (ii) Reducing bias

Suggested strategies for reducing bias (‘How do you think the Movement Ecology community could become less biased?’; Q5) ranged from interventions and actions targeted at the individual level, for example elevating researchers from under-represented backgrounds, to the institutional level, such as changing funding priorities (electronic supplementary material, table S3). Overall, public funding bodies, universities, scientific societies and individuals were most frequently mentioned as the entities that should be involved in reducing bias (55.8% of keywords, *n* = 88, ‘Which of the following entities should be involved in decreasing bias in the Movement Ecology community?’, Q6) (figure 1C). However, while there was consensus among groups with different experiences of bias that public funding bodies, scientific societies and universities should be involved in reducing bias, most individuals who had not personally experienced bias did not believe that individuals should be charged with reducing bias.

### (b) Observed bias

#### (i) Geographic bias for authors and research: *Movement Ecology* journal articles

**Figure 2.**
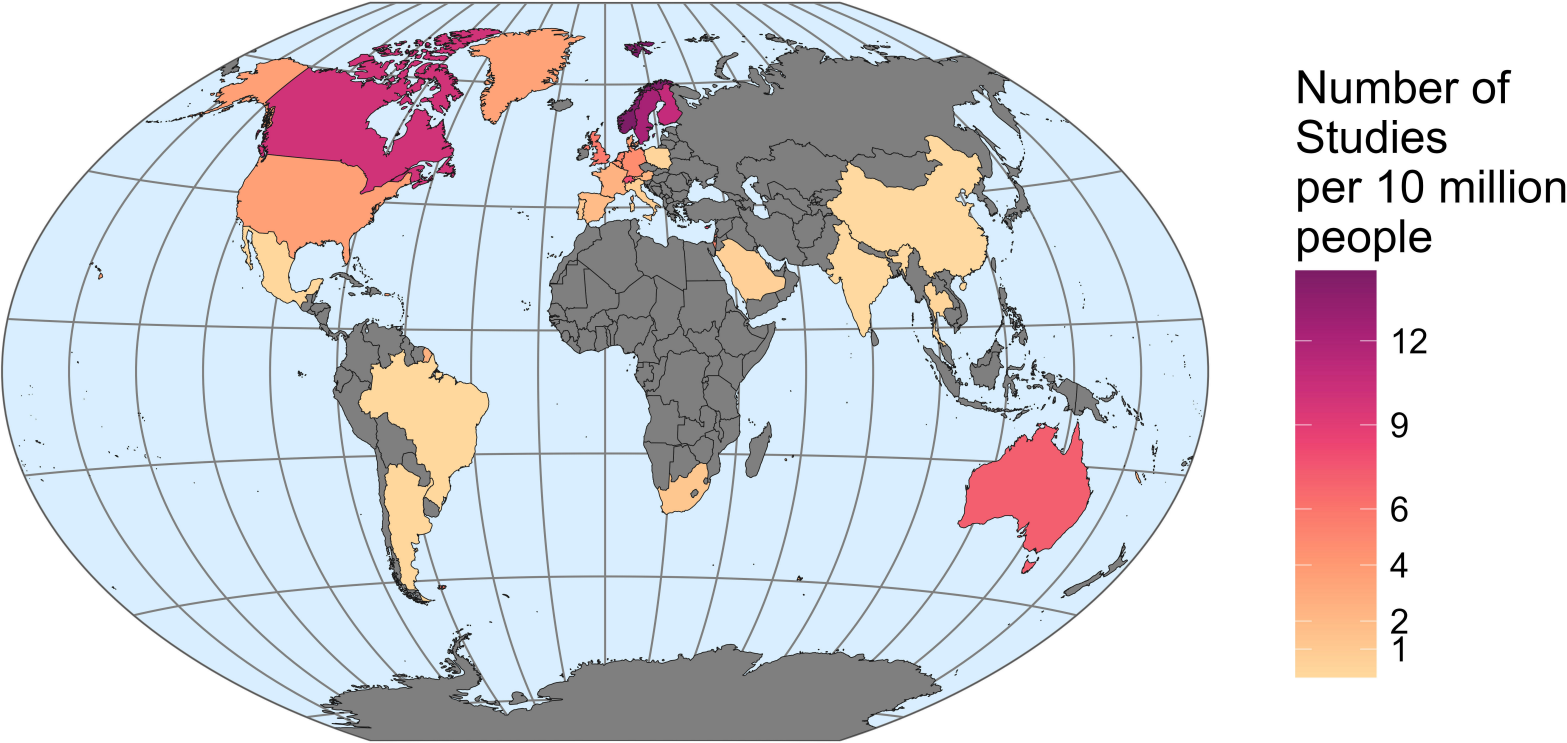
The number of times each country was listed as the first author’s first affiliation (standardized by country population size) for all 370 articles published in the journal *Movement Ecology* (from its launching in 2013 until January 2023). Colours indicate the number of studies per 10 million people; grey indicates no studies. See electronic supplementary material, figure S2 for corresponding author analysis.

**Figure 3.**
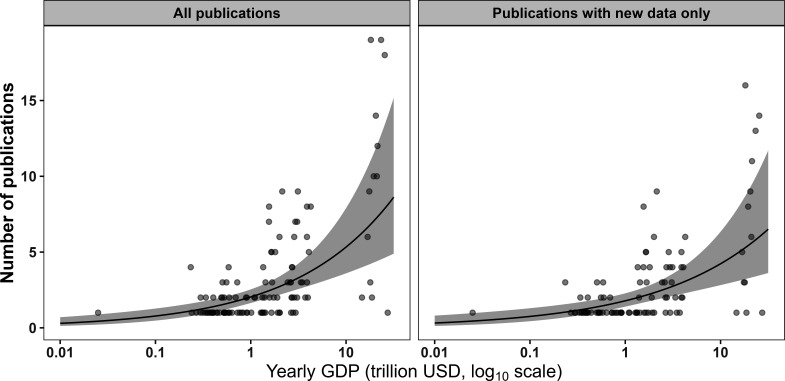
The relationship between a country’s gross domestic product (GDP) and the number of times it was listed as the first author’s first affiliation across (left) all 370 articles and (right) the 266 articles with new empirical primary data. Based on articles published in the journal *Movement Ecology* from 2013 to 2023. The black line indicates the estimated relationship, while the grey shaded areas indicate the 95% Bayesian credible intervals. See electronic supplementary material, figure S3 for per-capita comparison.

The analysis of geographic bias in research production showed that countries were not evenly represented (even controlling for population size) in authors’ national affiliations for articles published in the journal *Movement Ecology*. Only 28 countries (<15% of global countries) were represented by first authors in the journal (*n* = 370 articles; figure 2). There were 120 first authors affiliated with the United States of America, 40 with Canada (summing to 43% in North America), 40 with Germany and 39 with the United Kingdom (summing to 44% in Europe). All other countries were represented by less than 20 first authors (1% Latin America, 2% Middle East; 2% Africa, 2% Asia; 5% Oceania). The patterns were similar when looking at the country of affiliation of the corresponding author (electronic supplementary material, figure S2). Additionally, the average number of publications per year increased exponentially with yearly GDP (an indicator of the size of a country’s economy; figure 3, electronic supplementary material, figures S3–S5).

**Figure 4.**
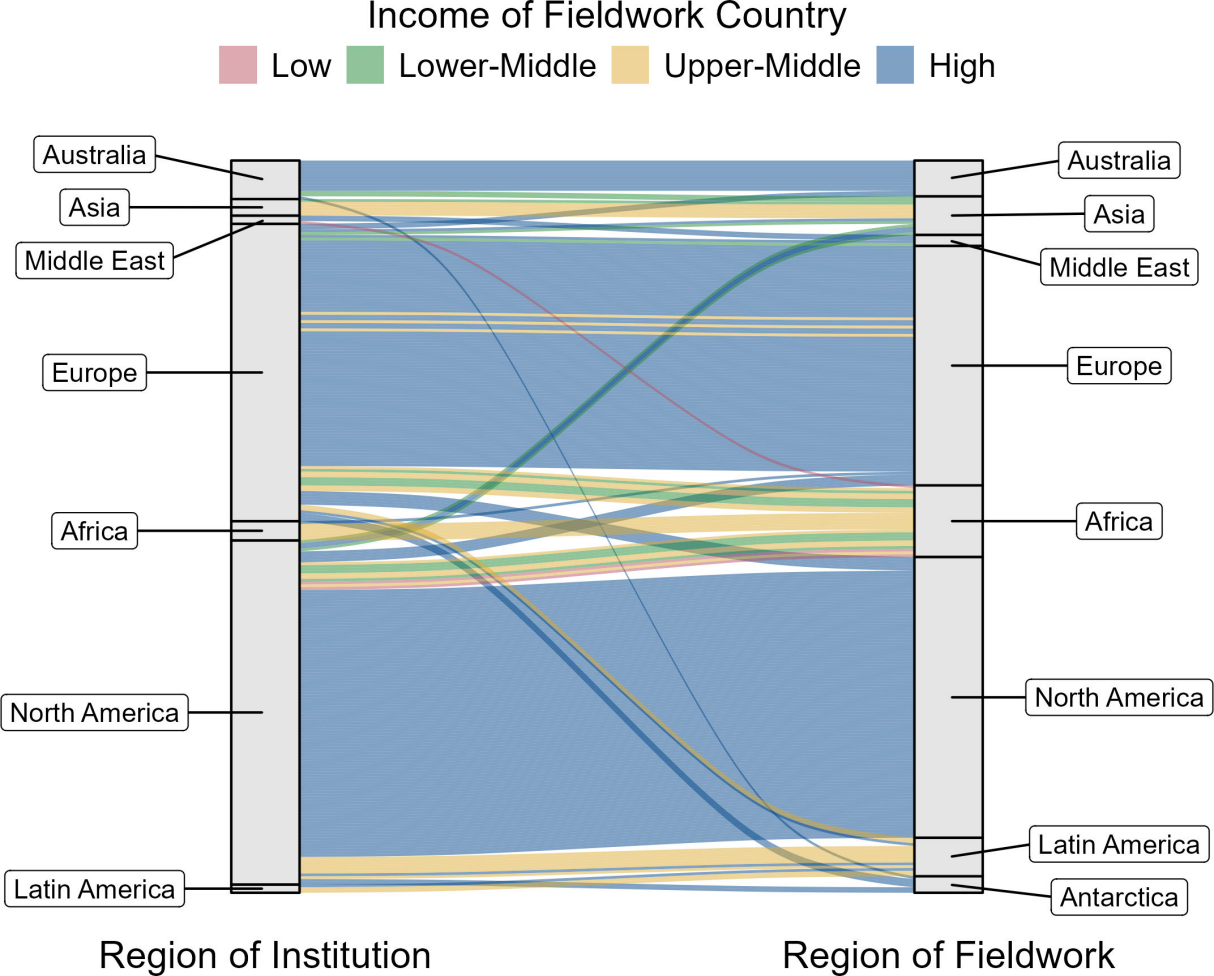
Comparison of first author’s regional affiliation (left) to region of fieldwork (right) for the 266 *Movement Ecology* studies (from 2013 to 2023) with new empirical data. Per-capita gross national income in 2022 (GNI) and regional categorization are from the World Bank: low (GNI <$1135), lower middle ($1136–$4465), upper middle ($4466–$13 845) and high (GNI > $13 846). See electronic supplementary material, table S4 for numbers.

Most studies were conducted in the same geographic region as the first author’s institutional affiliation. Of the 266 articles that collected new empirical data, 78% of studies conducted fieldwork in the same geographical region as the institution of the first author (figure 4, electronic supplementary material, table S4). For the 22% of the articles where the study site and first author affiliation were not identical, there were some notable disparities. A higher percentage of papers with first authors with institutional affiliations in the geographical regions of North America and Europe had fieldwork in other regions, compared to papers with first authors with institutional affiliations in Asia, Middle East, Africa and Latin America (electronic supplementary material, table S5). Most studies in Latin America were led by researchers based at institutions in North America. Most studies in Africa were led by researchers based in either North America or Europe with a fairly even split between the two. Furthermore, most studies that took place in low and lower-middle-income countries were led by researchers with institutional affiliations in other regions (figure 4, red and green bars). For studies in upper-middle income countries, the pattern varied by region—studies in Europe and Asia were led by researchers based in those same regions whereas studies in Africa and Latin America were led by researchers based in other regions (figure 4, yellow bars). However, most studies included an author with an affiliation in the country where data were collected (electronic supplementary material, table S6).

#### (ii) Race and gender bias in USA biology researchers

**Figure 5.**
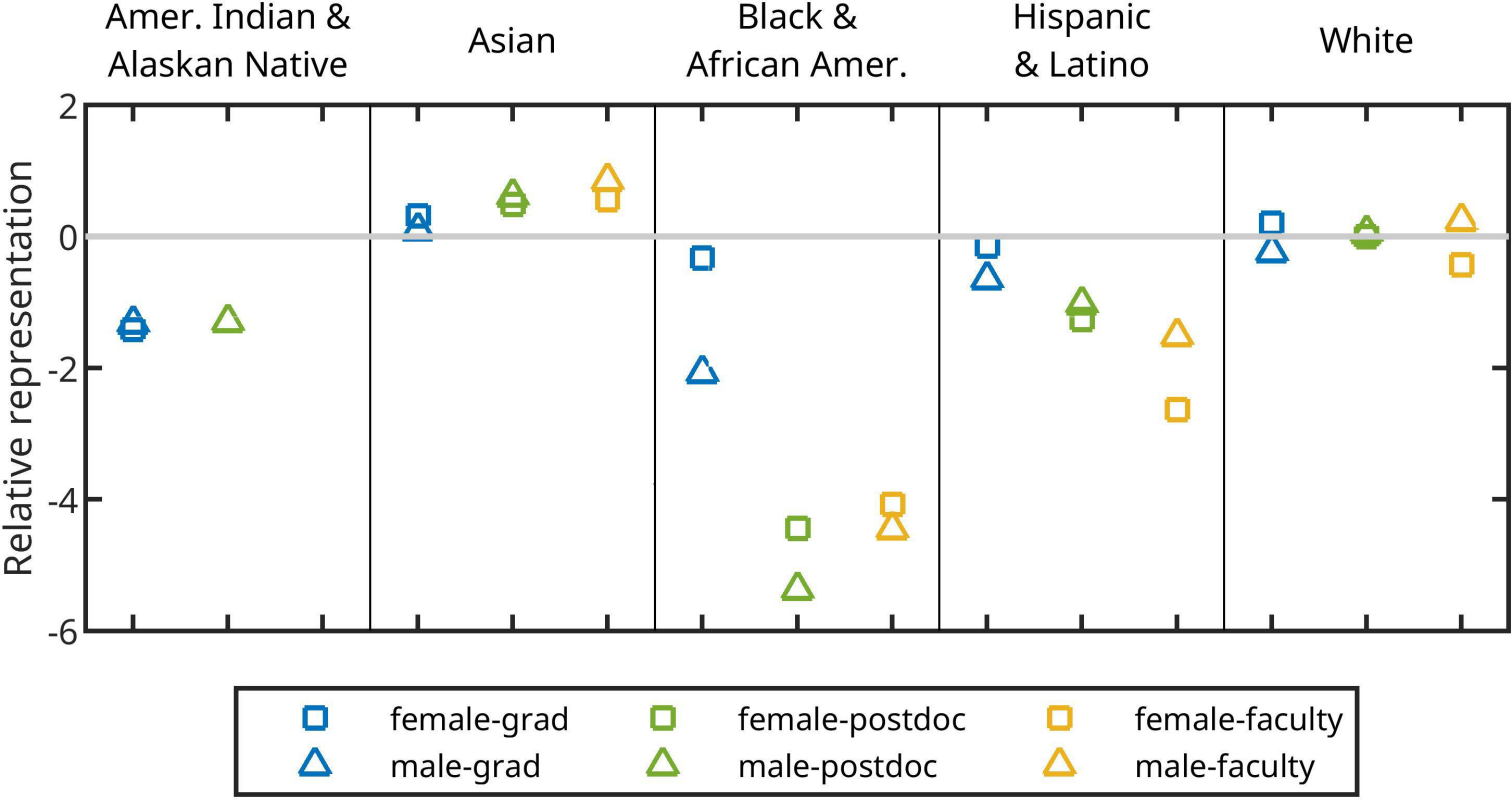
The relative representation of USA life science researchers across career stages. Positive values indicate overrepresentation compared to the USA population and negative values indicate underrepresentation. Race/ethnicity categories included American Indian and Alaska Native, Asian (including Pacific Islander), Black and African American, Hispanic and Latino, and White (see electronic supplementary material, S1). Gender categories included only female (squares) and male (circles). Data available on career-stages included graduate students (blue, but were only reported for USA citizens and permanent residents), postdoctoral researchers (green) and faculty (yellow).

In considering patterns of race/ethnicity and gender across career stages in biology, we found that representation of race-gender groups often differed from their representation in the general USA population (figure 5). White-male, Asian-male and Asian-female groups were all overrepresented among biology faculty compared to the general population, while all other race-gender groups were underrepresented. The most underrepresented race-gender group at all career stages was Black/African-American men, with underrepresentation generally increasing across career stages, and always lower than Black/African-American women. Within the race/ethnicity identities of White, Asian and Hispanic/Latino, women had higher representation than men among graduate students but lower representation at the faculty level.

### (c) Perceived bias informed by observed bias data: conference discussion

#### (i) In your words, what do you think bias is?

When asked to define bias, participants gave a variety of answers, describing bias as an intentional (explicit, conscious) or unintentional (implicit, unconscious) show of prejudice that can lead to systematic disparities in representation or opportunity. They described bias as encompassing outcomes and causes. Participants discussed that bias can result from misconceptions and subjective viewpoints that are not supported by evidence and may lead to differential treatment of some individuals. They suggested that people might focus on their own ideas, perspectives and experiences, tending to associate with other people who share similar identities or circumstances (e.g. socioeconomic status). Further views underlined that bias can also result from power imbalances (e.g. differences in academic rank), or confirmation bias. As a result, other legitimate viewpoints arising from different experiences or values are either ignored or rejected. They stated that biased perspectives limit opportunities for some individuals and can therefore result in reduced representation, leading to overall reduced diversity. And indicated that these disparities can have consequences that manifest in a variety of ways, from psychological well being (e.g. imposter syndrome or feeling unworthy) to research support (e.g. limited access to funding).

#### (ii) Before now, have you ever thought about bias in the movement ecology community?

Effectively all discussion participants reported having thought about bias within our community. The specific biases on which discussions focused spanned demographic, geographic and methodological. The community is aware of demographic biases that exist among its members. The conference attendees, the majority of whom are from (or are working in) Western nations (electronic supplementary material, figure S1), acknowledge that the sub-discipline continues to be dominated by white men, and that possibilities for career progression are not distributed equitably across gender and race identities. A significant point of concern was geographical bias due to the shaping of ideas, research paradigms and methodologies being predominantly driven by scientists in North America and Europe. The costs associated with publishing in open-access journals, attending international conferences and utilizing advanced research technologies (e.g. bio-logging devices) were thought to be major contributors of geographic bias through their disproportionate impacts on researchers, research topics and methodologies (e.g. sample size) from countries with lower GDP. An additional concern was the prevalence of ‘parachute or helicopter science’ where researchers from these regions descend on regions with lower GDP, often sidelining local scientists, failing to give credit to local communities and missing opportunities for skill transfer, local capacity building and knowledge exchange. Regarding research itself, biases affect methodology and topics. For example, research frequently favours popular or ‘charismatic’ species, while neglecting less well-known ones.

#### (iii) What is surprising or interesting to you about the survey results?

Multiple groups remarked on how closely the outcomes of the survey aligned with their expectations. Many of the groups discussed geographic biases underlying these results and were interested in how the data on the affiliations of first authors in the journal *Movement Ecology* would differ from the locations of data collection or the authors’ countries of origin. (Based on this discussion point, we extracted more information from the articles to make this comparison—described above, and presented in figure 4.) Some noted that the emphasis on geographic bias might be due to the composition of the survey pool, since many respondents referenced their own personal experience as evidence.

#### (iv) What is something you plan to do going forward?

Suggestions for actionable next steps ranged from changing individual perspectives, to exerting pressure to change community culture, to making needed policy changes to the academic system, and are presented below as statements provided during the discussion. Changing individual perspectives might require taking responsibility to acknowledge and mitigate one’s personal biases, identifying power dynamics, understanding the needs and the perspectives of others and acting on bias when witnessed. Several participants mentioned that it is hard to fight bias on their own, suggesting the need for collective action by peers and colleagues. Ideas for changes in behaviour differed across academic stage. More senior researchers indicated the need to involve people from the global community, to increase mentorship of underrepresented communities and to increase exchange opportunities for international students to obtain experience and training and to grow their professional networks. More junior researchers suggested the need to identify and reduce geographic and gender bias in citations used in academic publications. Participants suggested more outreach to show opportunities that are available in movement ecology, and starting diversity and equity groups (including reading groups) to raise awareness of bias in our sub-discipline. For peer-reviewing, considerations on the quality of the English writing should be separated from main comments (most journals offer a section for that), and constructive comments should account for potential sources of bias authors from underrepresented groups could be exposed to. Discussions highlighted that conference attendees were mostly native English speakers. Participants underlined that cheaper, more inclusive conferences are needed to promote change (e.g. hosting conferences in the Global South or offering formative opportunities like workshops). While some solutions, such as waiving conference or publishing fees, have sometimes been implemented, they are largely financial in nature and do not adequately address the full scope of the biases identified. For example, past efforts to increase the diversity of conference attendees by making funding available have fallen short (i.e. not many applications for conference scholarships from members of underrepresented groups are submitted). It was also noted that although each suggested action addresses a small part of the problem, together they will hopefully collectively improve inclusiveness within the community.

#### (v) What should we discuss doing as a community?

Participants discussed proposed solutions to bias captured by the survey results (figure 1C; electronic supplementary material, table S3). Many groups were frustrated that past anti-bias efforts, especially related to funding (e.g. funding agencies, peer-reviewed journal costs, conference fees), had been inefficient at creating desired change. For example, national funding agencies might only support conference attendance for scholars affiliated with institutions in that country. Several groups suggested encouraging senior researchers to leverage their role to do anti-bias work, such as by using relationships with tagging companies to secure more funding for scholarships. In terms of peer-reviewed publications, some comments were made about limitations associated with research costs and to what extent this should be accounted for by reviewers. Other solutions included the publication of abstracts in peer-reviewed papers in languages other than English, double-blind peer-review, focusing on inclusivity in invitations to publish in journal special issues and increased accessibility of data, code and tutorials. Similarly, choosing more affordable conference venues, considering visa issues or supporting virtual attendance may promote broader representation. Additionally, increasing the availability of funding targeted at increasing attendance by underrepresented groups could be beneficial; however, new ways to ensure increased attendance might need to be reconsidered (following results from past efforts). It was also recommended that the perception of exclusivity should be reduced, that rules around the diversity of chairs, speakers and panelists should be evaluated, and that each specific conference should rotate locations to increase accessibility from a greater diversity of geographic regions. Others suggested that in order to ensure that underrepresented communities are not disproportionately burdened with solving problems of bias, people from majority groups should increase their engagement in anti-bias work. Other proposed solutions included changes in attitudes, such as more listening and learning, i.e. making spaces more welcoming for underrepresented groups to speak up and be heard. It was also recommended that for future conferences in our sub-discipline we bring in a professional with expertise in diversity and equity to suggest actionable changes.

## 4. Discussion

Here, we studied bias in a scientific community, mainly by characterizing sources of observed and perceived bias within the sub-discipline of movement ecology as a case study. Our purpose was to explicitly bring a topic that is often implicit to the forefront, by quantifying patterns, understanding what factors shape views of these aspects within our community and providing motivation to broaden participation and inclusiveness in our sub-discipline. Below we first compare across our four sets of results, and then we situate the proposed solutions within existing literature on bias to assess whether potential solutions to resolve different types of bias that we identified had been previously implemented and their efficacy evaluated.

### (a) A community self-reflection on bias

We quantified *observed bias* in two different forms: country-level discrepancies in where authors publishing in *Movement Ecology* are based and where data are collected, and discrepancies in race/ethnicity and gender identity of biology academics compared to the general public of the USA. The first set of findings aligns with parachute science [42], which although sometimes supported by alleged good intentions, can fuel bias when not transformative beyond the single research project [43]. Also, it may indicate GDP-skewed research investments on this particular discipline. The second set of findings recovers some previously described results with respect to single axes of identity (i.e. that the representation of women decreases across academic stages and that Black/African-American and Hispanic/Latino identities are consistently under-represented within academia in the USA [12,44]). However, our findings also highlight places where considering multiple axes of identity together can provide new insights. Thus, care should also be taken in extrapolating our findings across axes. Our race-gender dataset is simultaneously more narrow (within the USA vs across countries) and more broad (all of biology vs movement ecology) than the geographies dataset, and biases present at one scale may not be present at others (e.g. [45]).

We examined *perceived bias* and found that it differed by context. For example, all conference discussion participants reported having previously thought about bias in movement ecology (informed perceived bias; which might be a reason why they decided to join the conference session), but our pre-conference survey showed a wide range of perceived degree of bias (uninformed perceived bias, figure 1A). Many of the themes raised in our discussions are aligned with those that have emerged in other discussions of STEM subdisciplines underpinned by fieldwork research [46,47]. Furthermore, the same sources of bias were not highlighted in both the survey and discussion. For example, both focused on geography, but the informed discussion focused less on race and gender. Specifically, for the open-ended survey question, the most common responses were gender/sexism and race/ethnicity/racism as key sources of bias (electronic supplementary material, table S2). However, for the multiple choice survey question, survey takers did not select ‘racism’ as a key source of bias, but they did select ‘BIPOC underrepresentation’ and ‘geographic concentration of researchers’. Survey participants who did select ‘racism’ as a key factor were exclusively those who drew on their personal experience to answer the survey (figure 1B). During the discussion (informed by the survey data), this pattern on sources of bias was amplified with little (if any) discussion of racism but extensive discussion of geographic and socioeconomic factors. One interpretation of these findings is differential comfort in discussing different sources of bias, especially in group settings.

### (b) Efficacy of solutions and future directions

Quantifying bias does little to move the conversation forward without a call to action. Survey takers suggested a number of actionable solutions (electronic supplementary material, table S3). To evaluate the effectiveness of these solutions, we conducted a preliminary literature search using the search terms from electronic supplementary material, table S3, along with additional keywords such as ‘STEM’, ‘bias’, ‘effectiveness’, ‘efficacy’, ‘reduce bias’ and ‘improve’ (electronic supplementary material, table S7). The most common solutions theme from our discussions related to personnel. These include strategies to elevate voices and engage groups, so that people with all perspectives/backgrounds are present where science is being discussed and conversations on how to strengthen our communities are taking place. Programs that improve advising/mentoring support [48] or increase training opportunities [49] have been successful. However, we could not find studies evaluating interventions to elevate researchers or address disparities in collaboration. The second most reported theme was financial. Programs that pay fair wages [50], or that offer seed grants or support for mentoring structures that promote retention [51] have been successful. Some financial interventions requiring systemic change on a national or global scale (e.g. redistribution of funding to address disparities across countries or by underrepresented groups) have not been tested, to our knowledge. The third theme was the scientific process—for example, changing the review process to avoid biases associated with author name and affiliation [52], or expanding perceptions of ecology beyond fieldwork to draw in more students [49]. In contrast, it is not clear whether open data policies—which lead to more citations [53]—also lead to reduced bias [53]. The fourth theme of solutions was events and actions. Past work has shown that workshops and courses can mitigate bias, but that the magnitude of change can vary with participant identity [54].

Overall, our investigation into the literature (electronic supplementary material, table S7) reveals that the scientific community has generated numerous proposals and suggestions to mitigate bias [55], including efforts to enhance diversity and eliminate barriers [49], and foster better access and equity [56]. A comprehensive review of the existing literature on bias reduction in movement ecology would require a new study; however, recent research by Meyer *et al.* [57] highlights successful diversity-increasing program interventions in STEM fields. Yet, these initiatives are not always implemented in (i) a testable format or (ii) documented within peer-reviewed journals. In instances where implementation has occurred, the degree of critical evaluation of its effectiveness has often been limited and frequently not conducted over extended timeframes. Furthermore, numerous interventions to reduce bias and improve diversity are short-term and rely heavily on self-reported data for assessment. As a community, we must take note of the insights shared by Meyer *et al.* [57] and devise future initiatives and programs, like those proposed here in electronic supplementary material, table S3, with a focus on thorough, long-term evaluations that keep in conversation with other sub-disciplines in the broader STEM community. Critically, we must include intervention assessment as part of the planning process, rather than as an after thought. Some actions could benefit from bringing in researchers from sociology, psychology and other specialized disciplines to provide structured recognition and mitigation of bias. For example, future work could consider how different forms of bias may affect one another or be correlated, and so contemporarily emerge.

Future research on quantitative studies of bias could follow up our work in a number of ways. First, one could survey a different subset of movement ecology researchers to assess their thoughts on bias, and how their perceptions relate to their identities, experience and context (by collecting demographic and other data from respondents—i.e. cross-checking multiple axes of bias at the same time). Our discussions of bias were surely shaped by the identities and experiences of conference participants (see positionality statement in electronic supplementary material, S1). Thus, a different set of movement ecologists may lead to a very different discussion about bias. Relatedly, one could explore the effect of priming that participants in the discussion experienced. Second, one could use a different classification scheme or look at a different spatial scale to consider the link between economic activity and publications. Further research should explore the disconnect we discovered between country affiliation of researchers and countries where the research is done. Although we focused on first and corresponding authors, one could test whether the patterns change by considering affiliation of senior authors. For papers with more than two authors, one could examine the diversity of middle authors’ affiliations, and whether it matches the affiliation of the first and last authors. This should be done with care as a higher diversity of countries of affiliation could be seen as positive (i.e. promoting international collaborations) or negative (i.e. relegating researchers who support local fieldwork as middle authors, rather than as leads). Author’s institution may not reflect an author’s country of origin, e.g. authors may pursue research in a wealthier country and use that support to conduct fieldwork in their country of origin. Research that surveys authors could determine how often this occurs. Some authors also had several affiliations from countries with different geographic and economic contexts, leaving unclear what portion of the research (e.g. data collection/analysis, PhD awarding, financial support) happened in which country. Second, future work could quantify biases in aspects of identity not addressed by our work, especially ones where safety and physical constraints can impede travel (e.g. LGBTQIA + identity, disability, caregiving).

The approach we used here could also be applied to other disciplines such as archaeology, geology and anthropology that obtain data with fieldwork. We could also compare our findings with other sub-disciplines of biology to see where biases are similar and different. For example, movement ecology may have more financial-driven bias than some sub-disciplines (e.g. by requiring expensive equipment, logistics and travel) or less than others (e.g. those that rely on large infrastructure such as genomics labs). Furthermore, fieldwork can have differential impacts on researchers based on the researcher’s identity [58], the interaction with stakeholders [59], or the cultural context.

## 5. Conclusion

Bias is omnipresent in science and we need to understand how biases develop and persist in order to proactively broaden representation. While presenting a partial view on bias in our community, we were able to identify some critical starting points that are particularly relevant to the sub-discipline (e.g. geographic and economic bias). While identifying concrete actions to address these drivers is at its infancy, we see a proactive attitude towards the risk of bias to be part of the solution. One advantage of our data-driven, community-based approach is that we can now monitor the impacts of this work at the community level (i.e. if and to what extent these conversations have been improving our community since their onset). Indeed, another key aspect we have identified is the lack of assessment for the actual implementation and effectiveness of possible bias-reducing solutions. This gap may decrease the awareness for bias and the drive towards concretely addressing it, as well as reduce lesson-learned opportunities. The availability of data on the composition of the community in its dissemination venues, such as journals, conferences or websites (e.g. https://ourworldindata.org/) and scientific societies, can help keep the attention on bias high, turning it into a factual rather than perceived or ‘personalized’ problem, and promote an evidence-based approach to its solution.

## Data Availability

The data and code can be found on Zenodo [60]. Supplementary material is available online [61].
